# Understanding the limitations of radiation-induced cell cycle checkpoints

**DOI:** 10.3109/10409238.2011.575764

**Published:** 2011-04-27

**Authors:** Dorothee Deckbar, Penny A Jeggo, Markus Lobrich

**Affiliations:** 1 Darmstadt University of Technology, Radiation Biology and DNA Repair, Darmstadt, Germany; 2 Genome Damage and Stability Centre, University of Sussex, East Sussex, UK

**Keywords:** Cell cycle checkpoints, double-strand break repair, ionising radiation, restriction point, genomic instability

## Abstract

The DNA damage response pathways involve processes of double-strand break (DSB) repair and cell cycle checkpoint control to prevent or limit entry into S phase or mitosis in the presence of unrepaired damage. Checkpoints can function to permanently remove damaged cells from the actively proliferating population but can also halt the cell cycle temporarily to provide time for the repair of DSBs. Although efficient in their ability to limit genomic instability, checkpoints are not foolproof but carry inherent limitations. Recent work has demonstrated that the G1/S checkpoint is slowly activated and allows cells to enter S phase in the presence of unrepaired DSBs for about 4–6 h post irradiation. During this time, only a slowing but not abolition of S-phase entry is observed. The G2/M checkpoint, in contrast, is quickly activated but only responds to a level of 10–20 DSBs such that cells with a low number of DSBs do not initiate the checkpoint or terminate arrest before repair is complete. Here, we discuss the limitations of these checkpoints in the context of the current knowledge of the factors involved. We suggest that the time needed to fully activate G1/S arrest reflects the existence of a restriction point in G1-phase progression. This point has previously been defined as the point when mitogen starvation fails to prevent cells from entering S phase. However, cells that passed the restriction point can respond to DSBs, albeit with reduced efficiency.

## Introduction

Cells are continuously exposed to endogenous and exogenous agents that damage DNA and compromise genomic integrity (Munro, 1970). To minimize the harmful impact of such continuous damage, cells have evolved DNA damage response (DDR) mechanisms that include DNA repair and processes regulating cell cycle progression (van Gent et al., 2001; Jackson & Bartek, 2009). The coordinated interplay of the DDR mechanisms is important to secure cellular fidelity and to avoid the development of chromosomal instability. A DNA double-strand break (DSB) is arguably one of the most deleterious lesions. Although DSBs arise less frequently than single-strand breaks (SSBs), they are potentially more significant. DSBs also arise following radiation exposure and, indeed, understanding the DDR process operating in response to DSBs is important for understanding the response to ionising radiation (IR). Several studies are aimed to investigate the interplay of DSB repair and cell cycle control mechanisms to understand how cell cycle progression is regulated in the presence of DSBs. From these studies, it became obvious that cell cycle control mechanisms exhibit limitations which can contribute to the development of genomic instability. For example, we and others have found that the G1/S checkpoint is not fully initiated until several hours post IR, allowing many cells to enter S phase with unrepaired DSBs and other damages (Linke et al., 1997b; Cann & Hicks, 2006; Deckbar et al., 2010). The G2/M checkpoint also has limitations, although they are distinct in nature, representing a failure to be activated after low doses (Deckbar et al., 2007; Krempler et al., 2007; Lobrich & Jeggo, 2007).

Here, we discuss the limitations of the G1/S and G2/M checkpoints in the context of the current molecular knowledge of DSB repair, cell cycle regulation and checkpoint control. We first provide a brief summary of the current knowledge of DSB repair, then discuss the processes that regulate cell cycle progression in an unperturbed cell and how DNA damage impacts upon these processes. Finally, we discuss the cellular and molecular mechanisms that underlie limitations to the damage-induced checkpoints.

## DSB repair mechanisms

DNA repair is the front line response to DNA damage. Eukaryotic cells have developed two main mechanisms to repair DSBs—nonhomologous end-joining (NHEJ) and homologous recombination (HR). NHEJ is active throughout the cell cycle and relies on rejoining free DNA ends without the need for sequence homology; HR, in contrast, involves the use of a homologous DNA sequence as a template for resynthesis. In yeast, the homologous chromosome can be used allowing HR to operate in all cell cycle phases. In contrast, in mammalian cells, HR only uses the sister chromatid restricting HR to the postreplicational cell cycle phases. However, even in G2, the majority of DSB repair occurs via NHEJ with HR being restricted to the repair of a subset of DSBs locating to heterochromatic regions (Beucher et al., 2009). In contrast to its limited role in G2, HR has a major role during S phase, both in repairing replication-associated DSBs and in promoting replication fork restart.

The detailed mechanisms of DSB repair have been the subject of several excellent reviews (Sancar et al., 2004; Helleday et al., 2007; Mahaney et al., 2009; Warmerdam & Kanaar, 2010). Briefly, during NHEJ, the free DSB ends are recognized and bound with high affinity by the Ku70/Ku80 heterodimer, which forms a ring-like structure enabling it to thread onto a DNA end, thereby protecting the ends from diffusing apart and undergoing nucleolytic degradation. Ku70/Ku80 stimulates the binding of the catalytic subunit of the DNA-dependent protein kinase (DNA-PKcs) to the outer end of the DNA which, together with Ku70/Ku80, constitutes the active DNA-PK holoenzyme. The subsequent *trans*-autophosphorylation of DNA-PK across the break site results in a conformational change and exposure of the DSB ends promoting recruitment of the Lig4/XRCC4/XLF complex and finally ligation. IR induces complex DSBs that possess additional lesions such as SSBs, base damages, abasic sites or phospho-glycosylates in close proximity to the DSB. Furthermore, chemicals such as topoisomerase II inhibitors and other drugs used for tumor treatment induce DSBs with covalently bound proteins or with single-stranded overhangs. In this case, the DSB is not directly ligatable and needs further processing prior to ligation. Such processing involves nucleases, such as Artemis, as well as additional enzymes that include polynucleotide kinase (PNK), DNA polymerases μ and γ and Werner's syndrome helicase (WRN; reviewed in Mahaney et al., 2009), which interact in concert with DNA-PK to remove these “bulky” lesions.

HR is initiated by the binding of the Mre11-Rad50-Nbs1 (MRN) complex to the free DSB ends. Subsequent resection is promoted by CtIP and the MRN complex and extended by Exo1 and Dna2 supported by the Bloom's syndrome helicase (BLM). However, the exact orchestration of nucleases and helicases in the course of resection remains unclear (Mimitou & Symington, 2009; Mazón et al., 2010). The resulting3'-overhangs, which can extend over several hundred bases, are stabilized by the single-strand binding protein, replication protein A (RPA), which is subsequently replaced by the recombinase Rad51. After homology searching, this nucleoprotein filament invades into the double-helix of the homologous sister chromatid. Using the free 3'-OH as a primer, DNA synthesis is performed by DNA-polymerase δ and η and ligation by DNA-Ligase I. The resulting Holliday Junctions are finally resolved by the helicase BLM in complex with TopIIIα/RMI1 (Cheok et al., 2005; Chu & Hickson, 2009). Recent studies have further identified Mus81-Eme,1 GEN1 and SLX1 as potential Holliday Junction resolvases (Ip et al., 2008; Fekairi et al., 2009; Muñoz et al., 2009; Svendsen et al., 2009; Svendsen & Harper, 2010).

## Cell cycle regulation and cell cycle checkpoints

The eukaryotic cell cycle consists of four phases and traversal from one to the other is regulated by Cyclins and Cyclin-dependent kinases (Cdks). Cyclins are small proteins that are expressed and degraded throughout the cell cycle in an oscillating manner. They gain their regulatory power by binding and activating Cdks, which phosphorylate a plethora of downstream substrates that regulate cell cycle progression.

DNA damage can be particularly harmful in certain cell cycle phases. For example, during S phase, DNA damage can interfere with replication fork progression and simple base damages can result in base mis-pairing causing point mutational changes. DNA-DNA or DNA-protein cross-links can lead to a distortion of the double-helix and also impede replication fork progression. Furthermore, unrepaired DSBs, SSBs and certain base damages can lead to replication fork stalling or collapse, which can compound the damage causing further DSBs and chromosome breaks. During cell division, chromosomal damage can result in loss of genetic material causing genetic alterations or even the death of the daughter cells. It is, therefore, not surprising that cells have developed tightly regulated mechanisms to control cell cycle progression in the presence of DSBs.

Following the induction of DNA damage, particularly DSB formation, cell cycle progression is interrupted to provide time for the removal of the damage. This is achieved by the activation of cell cycle checkpoints which target the Cyclin/Cdk complexes that normally promote cell cycle progression. Cell cycle checkpoints exist at the G1/S and G2/M boundary and are thought to prevent cells from replication or undergoing mitosis, respectively, in the presence of DNA damage. Furthermore, DSB induction can cause slowing of replication by locally inhibiting replication fork progression and new origin firing. Deficiencies in these S-phase checkpoints result in a radio-resistant DNA-synthesis (RDS) phenotype, i.e. cells are not able to stop synthesis in the presence of DSBs, which is a typical feature of cells from patients with chromosome instability syndromes such as ataxia telangiectasia (AT) or Nijmegen breakage syndrome (NBS; Falck et al., 2001, 2002). In contrast to these checkpoints that are directly induced by DNA damage, mitotic spindle checkpoints are activated indirectly by sensing the consequences of the damage, such as incorrect alignment at the equatorial plane and/or impaired formation or attachment of the spindle fibers at the kinetochores. In the following, we will focus on the regulation of the G1/S and the G2/M checkpoint.

## G1-phase progression and G1/S transition

**Figure 1:**
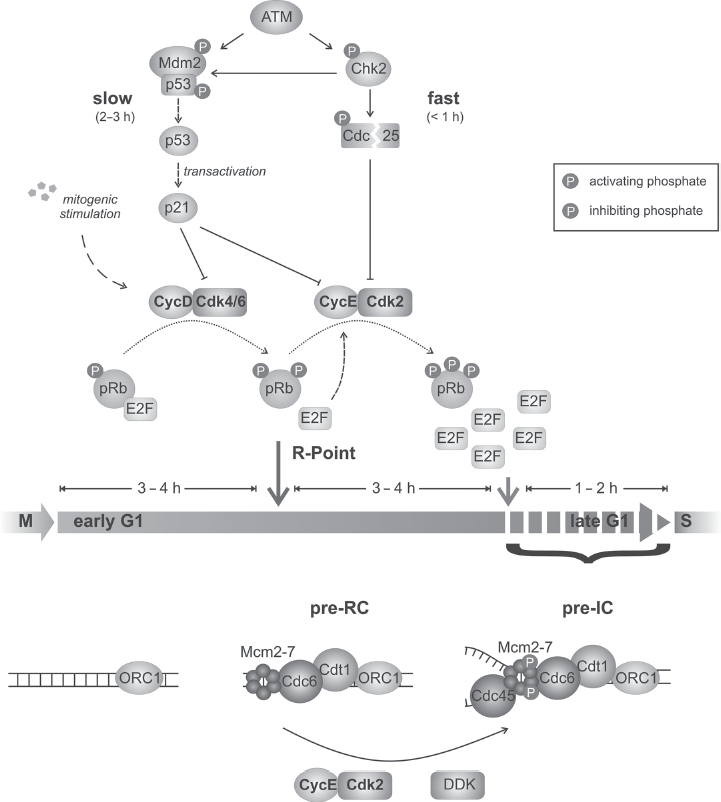
G1/S transition and G1/S checkpoint induction. The transition from G1 to S phase is initiated by the increasing mitogen-stimulated activity of CyclinD/Cdk4/6 which phosphorylates pRb resulting in the initial release of the E2F transcription factor. This activates a positive feedback loop rendering cell cycle progression independent of mitogenic stimulation, an event that has been termed *restriction point* (R-point; blue arrow). The feedback loop involves the upregulation of CyclinE which, in complex with Cdk2, further stimulates E2F release and the transcription of genes necessary for S phase. After full pRb phosphorylation, all E2F is released and cells are unable to respond to DNA damage via ATM-dependent checkpoint induction (red arrow). In parallel to pRb phosphorylation, replication origins are licensed and the pre-replication complex (pre-RC) is formed. Again, through the activity of CyclinE/Cdk2, Cdc45 is loaded onto the chromatin forming the pre-initiation complex (pre-IC) which is required for polymerase recruitment. After double-strand break (DSB) induction, two parallel checkpoint pathways target the activity of Cyclin/Cdk complexes (indicated in blue symbols). The slower pathway involves the stabilization of p53 and transcriptional upregulation of p21 which binds and inhibits the Cyclin/Cdk complexes. The faster pathway acts via the activation of Chk2 and the inactivation of Cdc25. Thus, inhibitory phosphates of the CyclinE/Cdk2 complex can no longer be removed. The indicated times are estimations and may vary considerably between cell types (see text for details). (See colour version of this figure online at www.informahealthcare.com/bmg)

The replication of the genome represents a process which is initiated soon after mitosis. At this point, mitogens stimulate the expression of CyclinD which associates with Cdk4 or 6, depending on the cell type. The resulting active CyclinD/Cdk4/6 complex has different targets, one of which is the retinoblastoma protein, pRb. In its hypophosphorylated state, pRb binds transcription factors of the E2F family which are required for cell cycle progression. As the level of CyclinD/Cdk4/6 complexes increases, pRb becomes phosphorylated and progression through G1 occurs. At a critical level of phosphorylation, E2F is released from pRb. This activates the transcription of CyclinE which complexes with Cdk2 to fully release pRb repression by further phosphorylation, establishing a positive feedback loop. E2F further promotes the transcription of S-phase genes. Thus, CyclinD/Cdk4/6 and CyclinE/Cdk2 together regulate S-phase entry via phosphorylating pRb, which controls pRb binding to E2F (Figure 1).

The restriction point is a critical stage during cell cycle progression at which the decision is made whether to undergo another round of replication or to transiently or permanently arrest in G0/G1. To characterize the response of cells at different positions in G1 to mitogen deprivation, 3T3 cells were serum-starved for 1h and the time taken to reach the next mitosis was measured (Zetterberg & Larsson, 1985). Cells that divided more than ∼3.5 h before serum deprivation did not show a significant delay in cell cycle progression, whereas cells that divided less than ∼3.5 h before serum deprivation displayed a delay of approximately 8 h. This important experiment demonstrated that there is only a short time window after cell division when the cell is responsive to external growth signals. The position in the cell cycle when the switch from mitogen-dependent to mitogen-independent cell cycle progression occurs was termed *the restriction point.* Along with this observation came the recognition that pRb is successively phosphorylated by Cdks during G1-phase progression and that the loss of pRb together with other E2F binding proteins abolishes the ability to enter a senescence-like state and increases the proliferation rate (Weinberg, 1995; Zetterberg et al., 1995; Dannenberg et al., 2000; Sage et al., 2000). Thus, today the restriction point is, in simple terms, the point in G1-phase progression when pRb phosphorylation exceeds a certain threshold level causing initial E2F release, which then activates the CyclinE/Cdk2 complex that leads to further pRb phosphorylation and more E2F release (Yao et al., 2008).

In addition to the successive phosphorylation of pRb by active Cyclin/Cdk complexes, other factors can also impact upon S-phase entry (Figure 1). For example, replication origins (ORI) have to be prepared (licensed) for replication initiation and a licensing checkpoint has been described (Ge & Blow, 2009; Nevis et al., 2009). ORI licensing starts in late mitosis/early G1 with the formation of the pre-replicative complex (pre-RC; Bell & Dutta, 2002; DePamphilis, 2003; Arias & Walter, 2007). Briefly, in mammalian cells, Orc 1 binds to ORIs at the M/G1 transition. This triggers independent recruitment of Cdc6 and Cdt1 and finally the Mcm2-7 complex. Disassembly of Orel, Cdc6 and Cdt1 from the chromatin ensues once Mcm2-7 is loaded (Rowles & Blow, 1997; Arias & Walter, 2007). Subsequently, the initiation of replication requires the formation of a pre-initiation complex (pre-IC) that is initiated by phosphorylation of Mcm2-7 by CyclinE/Cdk2 and DDK (Dbf4- and Drf1-dependent kinase) and recruitment of Cdc45 onto the chromatin (Figure 1). This recruitment is thought to be the critical step for the activation of the Mcm2-7 helicase activity and replication initiation. Finally, unwinding of the chromatin enables DNA-polymerase δ to initiate DNA synthesis and DNA-polymerase 8 to continue replication (reviewed e.g. in Bell & Dutta, 2002; Arias & Walter, 2007; Boye & Grallert, 2009).

To avoid re-replication of the same sequences, which would cause chromosomal instability, it is crucial that each origin only fires once. This is achieved by a range of mechanisms which, at least to some extent, are organism specific (reviewed e.g. in Arias & Walter, 2007). For example, the drosophila ortholog of Cdt1 is targeted for proteolysis following CyclinE/Cdk2-dependent phosphorylation. Thus, free Cdt1 is degraded once a cell has progressed to late G1 or S phase and CyclinE/Cdk2 becomes active (Thomer et al., 2004). In other multicellular organisms, Geminin has been identified to bind Cdt1 and prevent interaction between Cdt1 and Mcm2-7 (Wohlschlegel et al., 2000; Cook et al., 2004). At the transition from metaphase to anaphase, Geminin is ubiq-uitinated by APC^cdc20^ and degraded, allowing renewed origin licensing (McGarry & Kirschner, 1998).

## G1/S checkpoint induction and its limitations

**Figure 2:**
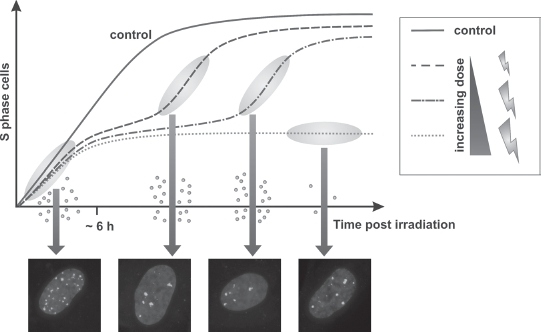
Limitations of the G1/Scheckpoint. The activation of the G1/S checkpoint is a slow but sensitive process. During the first 4–6 h after ionising radiation (IR), only a slowing of S-phase entry can be observed, allowing many cells to enter S phase even after high doses. Thus, during this time, many cells enter S phase with high double-strand break (DSB) levels. After full checkpoint activation, the G1/S checkpoint is sensitive to low DSB levels. The G1/S checkpoint is not necessarily permanent but cells can be released from it; the duration of arrest increases with dose. At the time of checkpoint release, most cells have slightly but significantly higher damage levels than unirradiated cells. After high doses, the checkpoint is maintained and only a few cells escape checkpoint arrest and enter S phase with elevated DSB levels. The figure depicts the S-phase entry of asynchronously irradiated cells. Edu is added prior to irradiation and cells in S phase at the time of irradiation are excluded from analysis (i.e. Edu^+^ cells). BrdU is added after irradiation and the figure shows the staining of BrdU^+^ Edu^−^ cells. (See colour version of this figure online at www.informahealthcare.com/bmg)

As stated above, the G1/S checkpoint is important to prevent damaged cells from entering S phase. To achieve this, the induction of DNA damage during G1 leads to the activation of signaling cascades which inactivate the CyclinD/Cdk4/6 and CyclinE/Cdk2 complexes that regulate S-phase entry. Two distinct mechanisms have been described (Figure 1; Iliakis et al., 2003; Lukas et al., 2004). One pathway involves the phosphorylation of p53 and its negative regulator Mdm2 by ataxia telangiectasia mutated (ATM) and Chk2 causing p53 activation and stabilization. p53 then transcriptionally upregulates the expression of target genes, of which p21 is critical for inhibiting G1/S entry. p21 is a Cdk inhibitor and binds CyclinE/Cdk2 and CyclinD/Cdk4/6 complexes. As this pathway involves transcriptional activation following posttranslational modifications, its full activation requires several hours and is assumed to be especially important for the maintenance of G1 arrest by inhibiting pRb phosphorylation. A second pathway involves only posttranslational modifications such as phosphorylations and ubiquitinylations and is therefore activated more rapidly. This response operates through ATM-dependent phosphorylation of Chk2 and subsequent destruction of the phosphatase Cdc25a, preventing the removal of inhibitory phosphates from Cdk2. Although studies have shown that Chk2-dependent regulation of Cdc25 can occur after damage in G1 phase (Bartek & Lukas, 2007), several studies have demonstrated that irradiation of middle or late G1-phase cells, even with unphysiologically high doses, does not abolish S-phase entry for 4–6h after IR (Gadbois & Lehnert, 1997; Linke et al., 1997b; Cann & Hicks, 2006; Deckbar et al., 2010). Thus, up to 4–6h post IR, only a slowing of S-phase entry is observed, even after high irradiation doses (e.g. 10 Gy; Figure 2). This, however, represents a defined damage response as it is abolished in the absence of ATM and Chk2.

These findings raise two questions; firstly, why does full G1/S arrest take 4–6h for activation; and, secondly, what is the cause for the slowing observed during this time. One possibility addressing the first question is that p21 activation requires posttranslational modifications and subsequent transcriptional activation and is, therefore, slow in its full activation. However, p21 expression can be observed within 2-3 h post IR suggesting that, although certainly a contributing factor, there may be another influencing factor. The concept of the restriction point is important in this context. Both the activation of the p53/p21 and the Chk2/Cdc25 pathways serve to inhibit Cdk activity; thus cells ahead of the restriction point may already have achieved sufficiently phosphorylated pRb that they are unresponsive or have limited sensitivity to respond to Cdk inhibition. It should, however, be considered that the restriction point defined by mitogen stimulation does not correspond to the point where cells can no longer respond to DNA damage, which is likely to be when pRb is fully phosphorylated following both CyclinD/Cdk4/6 and CyclinE/Cdk2 activation (see below). Thus, although the explanation is not entirely clear, it is evident that there is a point 4–6 h before S-phase entry after which cells cannot be fully inhibited for S-phase entry and that this is consistent with the notion of the timing of a restriction point and is not entirely explained solely by the time taken to activate p21.

In considering the mechanistic explanation underlying the second question, i.e. the early slowing in S-phase entry, it has to be appreciated that the cells that slowly enter S phase are those in late G1 phase. One possibility is that this process actually represents an S-phase checkpoint rather than a G1/S-phase checkpoint. Thus, cells may enter S phase but be slowed by the inhibition of origin firing. Although it is difficult to rule out the possibility that the slow S-phase entry correlates with slow S-phase progression, this was not supported by our analysis of the progression of early, mid and late S-phase cells (Deckbar et al., 2010). Thus, we favor the explanation that the ATM/Chk2/Cdc25-dependent slowing in S-phase entry represents a response of G1-phase cells—i.e. the arrest of a fraction of cells from entering S phase. Another possibility is that origin licensing is inhibited or slowed. As late G1-phase cells have already licensed many origins, this could result in slow S-phase entry if licensing is slowed. However, a radiation-induced damage response impacting upon licensing was reported to be independent of ATM and Chk2 (Higa et al., 2003), in contrast to the slowing observed in our studies (Deckbar et al., 2010). The prevailing evidence, therefore, suggests that within the first 4–6 h post IR, a Chk2/Cdc25 process is activated to inhibit Cdk activity akin to the process in G2/M phase.

It is striking that a small degree of slowing of S-phase entry can be observed after very low X-rays doses (100 mGy). Moreover, there is a clear dose response. Importantly, the observation of a dose response relationship above 1 Gy, a dose which can activate G1/S arrest in all cells after 4–6 h, argues strongly that there is a sensitivity difference between early and late G1-phase cells in their ability to undergo checkpoint arrest. Thus, the early Chk2/Cdc25 process is likely to be less sensitive at inhibiting Cdk activity than the late p53/p21 process, consistent with previous data (Buscemi et al., 2004). There are at least two possible explanations for the lower efficiency of the Chk2/Cdc25 process in comparison to the p53/p21 process. Firstly, in contrast to p21, inactiva-tion of Cdc25 inhibits the further activation of Cdks but does not inhibit Cdks which are already activated, potentially limiting its efficacy in late G1 phase. Secondly, the initial release of E2F at the restriction point defined by mitogen starvation initiates a positive feedback loop that increases Cdk2 activity which may be difficult to inhibit efficiently. Thus, both possibilities might result in a reduced but not abolished rate of pRb phosphorylation. However, the response appears unable to affect all cells as, even after high doses (10 Gy), only a modest slowing in S-phase entry is observed. This is difficult to explain solely by an inefficient Chk2/Cdc25 process and suggests that as cells near the point of S-phase entry, an increasing fraction of cells have traversed the point of full pRb phosphorylation and can no longer respond to DNA damage (NB: this point is different to the restriction point defined by mitogen starvation). Consistent with this, the magnitude of the response (i.e. the fraction of cells undergoing arrest) appears to diminish the closer the cells are to the time of S-phase entry. The existence of a time gap between full pRb phosphorylation and S-phase entry is also consistent with the notion that E2F, once released from pRb, transcriptionally activates factors needed for S-phase entry, a process which likely requires a significant amount of time.

In summary, during the first 4–6 h after IR exposure, there is a slowing but not full inhibition of S-phase entry and further studies are required to gain insight into the mechanistic explanation. Factors including differing sensitivity between the Chk2/Cdc25 and p53/p21 pathways, differing sensitivities of the cells behind and ahead of the restriction point and possibly differing durations between cells in their timing of entry after reaching the point of full pRb phosphorylation may contribute.

## G1/S checkpoint maintenance, release and chromosome break formation

As discussed above, several hours after IR, human fibroblasts display a full blockage of S-phase entry. This p53-dependent G1/S checkpoint is considered to be highly sensitive, possibly responding to a single unrepaired DSB (Di Leonardo et al., 1994; Wahl et al., 1997; Yamauchi et al., 2008). Despite this, chromosomal studies have shown that irradiation of p53-proficient G0/G1 cells can result in chromosome break formation in G2 or mitosis. Chromosome breaks (as opposed to chromatid breaks) are considered to arise when G0/G1-irradiated cells replicate across a DSB manifesting the breaks on both chromatids at the same location. How can this be explained if the G1/S checkpoint is responsive to a single DSB? Serum-starved cells that are irradiated immediately following serum addition undergo complete G1/S checkpoint arrest but can subsequently enter S phase (Deckbar et al., 2010). Under these conditions, the duration of checkpoint arrest was dose dependent and, after checkpoint release, the rate of S-phase entry was similar to that of untreated cells (Figure 2). Thus, the p53-dependent G1/S checkpoint not only functions to permanently arrest damaged cells but can also function transiently to enhance the time for repair (which we estimate to be ∼6h after 1 Gy). Interestingly, DSB repair measurements by γH2AX and 53BP1 foci analysis indicate that, although these cells had repaired the majority of DSBs when they commence S-phase entry, they had not completely reached the background damage level of unirradiated cells. Thus, the p53-dependent G1/S checkpoint is indeed very sensitive to low damage levels but cells released from it harbor unrepaired damage (possibly 1–3 DSBs). Additionally, after high doses, when the majority of cells undergo prolonged (and possibly permanent) arrest, a small fraction of cells can escape arrest, enter S phase and progress to G2 phase with substantial levels of unrepaired damage. It is possible that the analysis of mitotic chromosome breakage after high doses selects for such cells.

The fact that chromosome breaks arise after IR in G0/G1 also requires a consideration of the sensitivity of the S-phase checkpoints. It is generally assumed that chromosome breaks (as compared to chromatid breaks) are the outcome of a DSB that has been duplicated in the course of replication. This implies that the intra-S checkpoint is inefficient in preventing replication of broken DNA. This is most obviously demonstrated by the observation that S-phase cells irradiated with high doses are able to complete replication within a few hours. Furthermore, irradiated S-phase cells enter mitosis at similar times to irradiated G2-phase cells, indicating that the intra-S checkpoint does not provide any significant additional repair time (Deckbar et al., 2007; Krempler et al., 2007; Fernet et al., 2010).

## G2/M transition

**Figure 3:**
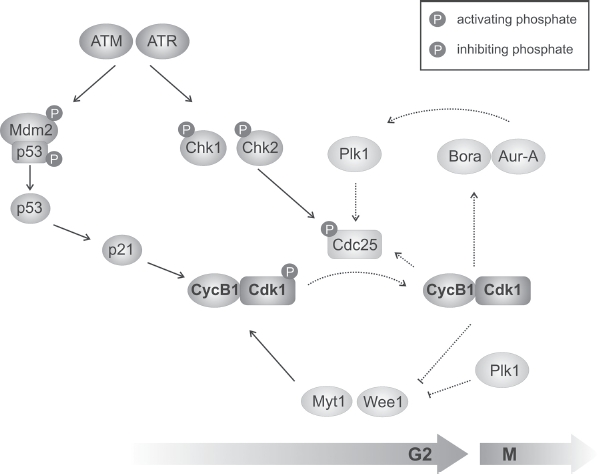
G2/M transition and G2/M checkpoint induction. Entry into mitosis is triggered by an active CyclinB1/Cdk1 complex. The activity of the CyclinB1/Cdk1 complex is tightly regulated by opposing feedback loops (indicated in green)—one involving the kinases, Myt1 and Wee1, and the other the phosphatase Cdc25, the first phosphorylating and inhibiting CyclinB1/Cdk1 and the latter removing these phosphorylations. Plk1 is involved in both feedback loops. After DNA damage induction, two ATM-dependent checkpoint pathways are activated (indicated in blue). The checkpoint kinases, Chk1/Chk2, target Cdc25 for nuclear export leading to the accumulation of the inactive CyclinB1/Cdk1 complex. Further inhibition of CyclinB1/Cdk1 takes place through a slowly activated p53-dependent pathway. The lack of activated CyclinB1/Cdk1 interrupts the feedback loops (dotted lines), resulting in G2 arrest. (See colour version of this figure online at www.informahealthcare.com/bmg)

The protein complex that drives mitotic entry is CyclinB1/Cdk1. Transcription factors regulating CyclinB1 are activated by Cdks ensuring that transcription of CyclinB1 only takes place in the presence of active CyclinA/Cdk2. Thus, transcription of CyclinB1 starts in S phase and peaks in late G2 (reviewed in Fung & Poon, 2005). In mammalian cells, Cdk1 levels are generally higher than CyclinB1 levels. Thus, the presence of CyclinB1 and, therefore, the possibility to form a complex is the limiting factor for mitotic entry (Arooz et al., 2000). During G2, the CyclinB1/Cdk1 complex is held in an inactive state through phosphorylation of Tyr15 and Thr14 by the Wee1 and Myt1 kinases (Nigg, 2001). For activation and entry into mitosis, dephosphorylation of Cdk1 by the Cdc25 phosphatases is required. Thus, the regulation of CyclinB1/Cdk1 is performed by the opposing activities of Wee1 and Cdc25 (Figure 3).

In interphase cells, CyclinB1/Cdk1 is retained in the cytoplasm through permanent nuclear export. Located at the centrosomes, it is held in an inactive state by Chk1 -dependent inhibition of Cdc25 to prevent unscheduled cell division (Krämer et al., 2004; Loffler et al., 2006). Centrosomally located CyclinB1/Cdk1 is initially activated at the centrosomes shortly before they start to migrate apart and rapidly translocates into the nucleus (Hagting et al., 1998; De Souza et al., 2000; Jackman et al., 2003; Loffler et al., 2006). Once activated, CyclinB1/Cdk1 phosphorylates Wee1 and Cdc25, inactivating Wee1 and activating Cdc25, which causes canonical activation of further CyclinB1/Cdk1 complexes. Thus, by inactivating its own inhibitor and activating its activator, CyclinB1/Cdk1 is the central component of a positive feedback loop that ensures full and committed mitotic entry (reviewed in Lindqvist et al., 2009; Figure 3).

The activation of Cdk1 has wide-ranging consequences. Firstly, activated Cdk1 phosphorylates Wee1 which mediates Plk1 recruitment and subsequent Plk1-dependent phosphorylation of Wee1. The dual phosphorylation of Wee1 (i.e. Cdk1 and Plk phosphorylations) results in its poly-ubiquitination and proteasomal degradation (Watanabe et al., 2004; Watanabe et al., 2005). In parallel, Plk1 phosphorylates Cdc25 contributing to its nuclear accumulation. Recent studies demonstrated that the initial phosphorylation of Plk1 at the G2/M transition is conducted by Aurora-A kinase (Macurek et al., 2008; Seki et al., 2008; Macurek et al., 2009). Activation of Aurora-A requires Bora which is also activated by Cdk1. Thus, Cdk1 phosphorylates Bora leading to further activation of Plk1 (Hutterer et al., 2006; Chan et al., 2008). Thus, in a second feedback loop, CyclinB1/Cdk1 stimulates its own activation by enhancing the activity of its own activators. Furthermore, dephosphorylation of Aurora-A by PP1 results in its inactivation (Katayama et al., 2001; Marumoto et al., 2002). Here, again, CyclinB1/Cdk1 affects its own activation by regulating PP1 activity. Having reached a certain level in late G2/early mitosis, CyclinB1 contributes to the activation of the multisub-unit E3 ligase APC/C promoting its own proteosomal degradation ensuring low expression levels at the beginning of the new cell cycle (Fung & Poon, 2005; Lindqvist et al., 2007).

In summary, a whole network of positive and double-negative feedback loops exists for the activation of CyclinB1/Cdk1. It has been proposed that this activation occurs continuously during the progression through S and G2 phase. The feedback loops warrant that after CyclinB1/Cdk1 is activated above a certain threshold level, further activation increases rapidly and initiates the subsequent cell division.

## G2/M checkpoint activation

Before CyclinB1/Cdk1 has reached a critical level, cells can respond to DNA-damaging agents by interfering with the feedback loops that lead to further activation of CyclinB1/Cdk1. DSBs, for example, activate ATM which phosphorylates effector proteins and initiates cell cycle arrest. The molecular mechanisms are very similar to those of the G1/S checkpoint and have been presented in various reviews (Lukas et al., 2004; Sancar et al., 2004; Warmerdam & Kanaar, 2010). Briefly, active ATM phosphorylates Chk2 which, in turn, phosphorylates Cdc25, leading to cytoplasmic translocation of Cdc25 and maintained inhibition/inactivation of CyclinB1/Cdk1 (Figure 3). Thus, this pathway consists of posttranslational modifications resulting in rapid G2 arrest. Although a p53-dependent pathway which involves the transactivation of Cdk-inhibiting proteins has been described, the exact function of this pathway in regulating G2/M arrest is not fully understood (Chan et al., 2000; Taylor & Stark, 2001; Lukas et al., 2004; Bruno et al., 2006).

One function of a checkpoint is to halt cell cycle progression until the completion of DSB repair or until the environmental conditions are optimal for cell cycle progression (and cell division in the case of the G2/M checkpoint). Nevertheless, a phenomenon has been reported that allows cell division in the presence of unrepaired DSBs. This process was originally described in *Saccharomyces cerevisiae* and demonstrates that yeast cells carrying a dispensable chromosome without a telomere (which represents a DSB) first undergo G2 arrest but subsequently abrogate the checkpoint and undergo division (Sandell & Zakian, 1993). This process was termed *adaptation* and has been suggested to cause genomic rearrangements and genomic instability (Sandell & Zakian, 1993; Toczyski et al., 1997; Galgoczy & Toczyski, 2001). A unicellular organism, which has to adjust to changing environmental conditions, may benefit from this but *adaptation* has also been demonstrated in *Xenopus* egg extracts (Yoo et al., 2004) and has been suggested to exist also in human cells. Indeed, using G2/M checkpoint-proficient tumor cells, it has been demonstrated that tumor cells can divide in the presence of unrepaired DSBs several hours after IR and G2/M checkpoint activation (Syljuåsen et al., 2006; Syljuåsen, 2007).

**Figure 4:**
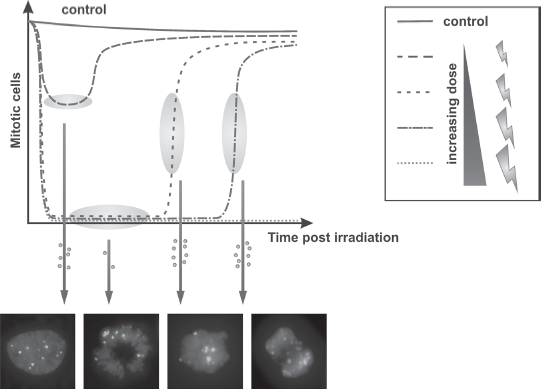
Limitations of the G2/M checkpoint. The activation of the G2/M checkpoint is a fast but insensitive process. Low-dose irradiation fails to completely prevent entry into mitosis resulting in many cells entering mitosis with double-strand breaks (DSBs). Higher doses (≥0.5–1 Gy) fully initiate the G2/M checkpoint with only a few cells escaping arrest. The duration of the G2 arrest increases with dose. However, the G2/M checkpoint is not maintained until the completion of DSB repair but cells are released when they harbor 10-20 γH2AX foci. This limitation results in many cells entering mitosis with a substantial amount of unrepaired DSBs. The figure depicts entry into mitosis of asynchronously irradiated cells. pSer 10-H3 was used to assess mitotic cells at various times post irradiation. (See colour version of this figure online at www.informahealthcare.com/bmg)

Additional insight into G2/M checkpoint regulation after IR was provided by recent studies with normal human fibroblasts which demonstrated that mitotic entry occurs prior to the completion of DSB repair (Deckbar et al., 2007). Thus, mitotic entry in the presence of unrepaired DSBs is not restricted to tumor cells but represents a physiological process that occurs even in nontransformed cells. However, examination of the kinetics of mitotic entry after different doses in repair-proficient as well as repair-deficient cells demonstrated that cells are released from G2 arrest once their level of DSBs has fallen below a certain threshold. Surprisingly, this threshold is not a single DSB, the level necessary to arrest cells until the completion of DSB repair, but rather the G2/M checkpoint is abrogated in the majority of cells when they harbor between 10–20 unrepaired DSBs (Figure 4). The finding of a defined threshold was surprising and appeared to be inconsistent with chromosomal studies reporting a dose-dependent increase in mitotic chromosome breakage. However, these studies assess the level of chromosome breakage at a defined time point post irradiation and do not consider the response of the majority of cells that undergo checkpoint arrest. Indeed, most cells undergo arrest and are released within a similar time frame, with the duration of arrest being dose dependent. Thus, at any defined time point post irradiation, the level of chromosome breaks increases with dose, but at the specific time when the majority of cells enter mitosis (i.e. when checkpoint arrest is released), the number of chromosomal breaks in mitosis is dose independent (Löbrich & Jeggo, 2005; Jeggo & Löbrich, 2006).

In summary, the G2/M checkpoint transiently arrests heavily damaged cells in G2 to provide time for repair but does not completely prevent cell division in the presence of unrepaired DSBs, allowing cells to enter mitosis with a significant number of DSBs (Figure 4). Thus, although being rapidly activated (in contrast to the G1/S checkpoint), the G2/M checkpoint also has inherent insensitivity.

## G2/M checkpoint termination

In contrast to the number of studies addressing the mechanisms of checkpoint induction, little is known about the biochemical mechanisms underlying the termination of G2 checkpoint arrest and the resumption of cell cycle progression. More recently, however, checkpoint termination became the subject of several investigations. In principle, there are two distinct mechanisms underlying checkpoint termination. Protein phosphatases such as Wip1, PP2A, PP4 or PP6 counteract the activity of the checkpoint activating kinases, ATM and ATR, by removing phosphates from Chk1, Chk2, p53 and the damage signal amplifying histone variant H2AX (Goodarzi et al., 2004; Lu et al., 2005; Shreeram et al., 2006; Nakada et al., 2008). In the course of repair, the checkpoint activating signal decreases shifting the balance between activating and inactivating signals toward inactivation. Once the cell has repaired below a certain DSB level, phosphatase activities dominate over the kinase activities and boost the process of checkpoint termination. Indeed, depletion of these phosphatases results in impaired checkpoint termination. The second possibility underlying checkpoint termination involves the activation of pathways during checkpoint arrest which counteract the checkpoint activation pathways. The first studies which shed light on the existence of such active pathways were again performed in yeast. The *adaptation* process described above depends on at least three genes: the polo-like kinase Cdc5 and two genes encoding the casein kinase II subunits Ckb1 and Ckb2 (Toczyski et al., 1997). The single-stranded DNA (ssDNA) binding protein, RPA, as well as Ku70, Mre11 and Rad50 which affect the generation of ssDNA, were also suggested to regulate checkpoint adaptation (Lee et al., 1998). Studies using replication stress for DSB induction in aphidicolin-treated *Xenopus* egg extracts extended the list of potential key players in the process of *adaptation* to DNA damages to Plx1 (human Plk1), Claspin and Chk1 (Yoo et al., 2004). Aphidicolin-treated egg extracts initiate a G2/M checkpoint response and undergo adaptation after several hours. Claspin was originally described as a Chk1-interacting protein. During replicational stress (which occurs after aphidicolin treatment), Claspin binds to chromatin and is essential for the phosphorylation and activation of Chk1 in *Xenopus* and human cell systems (Kumagai & Dunphy, 2000; Chini & Chen, 2004). Claspin becomes phosphorylated at Thr906 in an ATR-dependent manner, which creates a docking site for Plx1 enabling Plx1 to phosphorylate Claspin on Ser934. In the course of checkpoint adaptation, despite the persisting replicational stress, Claspin is progressively phosphorylated in a Plx1-dependent manner, resulting in the release of Claspin from the chromatin and finally the inactivation of Chk1 (Yoo et al., 2004).

The events leading to checkpoint termination are even more complex as the ubiquitin ligase SCF-βTrCP regulates two opposing effects. On one hand, SCF-βTrCP ubiquitinylates Cdc25a triggering its degradation, resulting in checkpoint activation after replicational stress. On the other hand, it ubiquitinylates Claspin resulting in Claspin degradation and thus Chk1 inactivation (Mailand et al., 2006). Again, the switch from a cell cycle inhibitory to a cell cycle promoting mode of βTrCP has been suggested to be mediated by Plk1 which phosphorylates the N-terminus of Claspin promoting its SCF-βTrCP-dependent ubiquitinylation and degradation. Furthermore, Plk1 triggers βTrCP-dependent ubiquitinylation and degradation of the Cdk1-inhibiting kinase Wee1.

The studies above address G2/M checkpoint termination after replication stress involving ATR, Chk1 and Wee1. However, DSBs are primarily sensed by ATM and Chk2. Currently, little is known about G2/M checkpoint termination after DSB induction. It has recently been demonstrated that Cdk1 phosphorylates 53BP1 at Ser380, a highly conserved Plk1 *Polo-Box Domain* (PBD)-binding site (van Vugt et al., 2010). Upon phosphorylation, Plk1 binds 53BP1 which serves as a binding platform for Chk2. Subsequently, Plk1 phosphorylates the *forkhead-associated* (FHA) domain of Chk2 which results in Chk2 inactivation. Furthermore, 53BP1 (as well as pATM) does not form DDR foci during mitosis preventing sustained damage signaling. In this way, 53BP1 could play a central role in checkpoint termination, with phosphorylation of 53BP1 impacting on both the efficient maintenance of ATM signaling and the downstream signaling via Chk2. However, there are difficulties with the model that phosphorylation of 53BP1 promotes checkpoint termination. Firstly, it is unclear whether the release of 53BP1 from the DSB site is a consequence or the cause of checkpoint termination. We have observed that 53BP1 foci can be detected reliably until early prophase and that 53BP1 release from chromatin occurs concomitantly with chromatin condensation (unpublished data). This would imply that 53BP1 release from the chromatin does not promote checkpoint termination but rather represents the consequence of chromatin condensation during mitotic entry. A second difficulty is that cells lacking 53BP1 undergo inefficient checkpoint arrest at low doses and premature checkpoint release after high doses, i.e. the opposite of what would be predicted from a model in which 53BP1 is required for the termination of checkpoint arrest (Fernandez-Capetillo et al., 2002; Shibata et al., 2010). Finally, in this context, it is worth mentioning the role of γH2AX foci formation in both the initiation and maintenance of checkpoint arrest. Cells lacking H2AX are able to effect checkpoint arrest at all but low IR doses (Fernandez-Capetillo et al., 2002; Shibata et al., 2010). They also fail to maintain arrest efficiently. The findings suggest that the foci have an amplification role which is solely required at low doses. Surprisingly, despite this process to enhance signaling at low doses, cells remain insensitive to G2/M checkpoint arrest when less than 15–20 DSBs remain.

The transition from G2 into mitosis is a tightly regulated process consisting of positive and negative feedback loops which target the activity of the CyclinB1/Cdk1 complex, and it is likely that entry into mitosis occurs when the activating signals dominate over the inhibitory signals.

## Interplay between DSB repair in G2 and the G2/M checkpoint

The G2/M checkpoint signal is essentially dependent on active Chk1 and Chk2 as the addition of Chk1/2 -inhibiting drugs after checkpoint initiation results in abrogation of the G2 arrest within 1h (Shibata et al., 2010). However, G2/M checkpoint signaling can be modified during DSB repair. In G2-irradiated cells, about 20% of IR-induced DSBs are repaired with slow kinetics representing those DSBs that undergo resection and repair via HR (Beucher et al., 2009). Resected ssDNA regions are rapidly coated with RPA leading to a switch from ATM to ATR activation (Zou & Elledge, 2003; Shiotani & Zou, 2009). However, as resection is dependent on ATM, ATR activation and G2/M checkpoint induction relies on ATM Jazayeri et al., 2006). ATM and ATR predominantly signal to Chk2 and Chk1, respectively. As resection can be initiated at early times post IR, Chk1 and Chk2 have overlapping roles in checkpoint initiation (López-Contreras & Fernandez-Capetillo, 2010; Shibata et al., 2010). At early times post IR, the majority of DSBs are unresected; thus, it is possible, although it has not been definitively shown, that ATM/Chk2 plays the major role in checkpoint initiation. However, Chk2^-/-^ mouse embryonic fibroblast cultures (MEFs) are proficient in checkpoint induction, suggesting that Chk1 can fully compensate for the loss of Chk2 (Fernandez-Capetillo et al., 2002). At later times post IR, the ratio of resected versus unresected DSBs increases as the repair of unresected DSBs by NHEJ is a fast process. This, in turn, results in a greater contribution of ATR and Chk1 to the maintenance of checkpoint arrest. Thus, ATR/Chk1-deficient cells exhibit premature G2/M checkpoint release (Shibata et al., 2010).

Given that cells are released into mitosis before the completion of DSB repair, it is important to consider the consequences of this limitation. One consequence is the appearance of chromosomal breaks in mitosis which can lead to loss of genetic material and cell death. Additionally, as mentioned above, HR represents the slow component of DSB repair in G2 (Beucher et al., 2009). This suggests that at the time of checkpoint release, the remaining DSBs represent those undergoing repair by HR. Thus, it is possible that cells enter mitosis with resected DSBs as well as with hetero-duplexes and unresolved Holliday Junctions. Anaphase bridges are microscopically visible chromatin connections between the chromatids of the two daughter cells and represent markers for chromatin entanglements (Hoffelder et al., 2004). If not resolved in time, anaphase bridges can break in later stages of mitosis and lead to the *de novo* formation of DSBs. Observations from our lab indicate that X-irradiation of G2-phase cells increases the number of anaphase bridges in mitosis (unpublished data). Thus, another consequence of the insensitivity of the G2/M checkpoint could be the formation of anaphase bridges which result in the formation of *de novo* DSBs and micronuclei. Recent observations strengthen these suggestions by demonstrating that cells entering mitosis with DNA damages induced by replicational stress displayed elevated levels of 53BP1 foci in the following G1 phase (Lukas et al., 2011).

## The cooperation of cell cycle checkpoints in the prevention of chromosomal instability

As discussed, cell cycle checkpoints have significant limitations. The G1/S checkpoint fails to prevent cells with DSBs from entering S phase within the first hours after irradiation. Such cells exhibit significantly elevated levels of unrepaired DSBs in G2 as well as chromosome breaks. As the G2/M checkpoint is insensitive below 10–20 DSBs, cells undergo mitosis with unrepaired DSBs which can result in loss of genetic material. Thus, neither the intra-S nor the G2/M checkpoint can compensate for the limitation of the G1/S checkpoint. However, it has been observed that following one round of cell division, cells irradiated in G1 are temporarily or permanently arrested in the following G1 phase (Linke et al., 1997a; Wahl et al., 1997; unpublished data). Interestingly, this was the case for cells that had been irradiated in late G1 and failed to arrest as well as for cells that had initiated and subsequently been released from the G1/S checkpoint. This second round arrest process is p53 dependent suggesting that the most prominent role of p53 might be the regulation of DNA damage-activated processes over multiple cell cycles. It is possible that this mechanism eliminates cells with irreparable damage from the actively proliferating population (Wahl et al., 1997; Petersen et al., 2010). Multicellular organisms aim to achieve a balance between cell survival and the risk of developing chromosomal instability. DSB-activated checkpoints generally provide time for repair but there appears to be a need that they are limited in their efficiency. It has to be appreciated that even in an unperturbed cell cycle, DSBs are induced by products of the oxidative metabolism, during the course of replication and, in some cell types, in a programmed manner. One possible explanation for the need for inefficiency is that if checkpoints were too tight, the risk of developing chromosomal instability would be minimized but at the cost of limiting the ability of cells to proliferate, and consequently the survival of an organism. However, if checkpoints were too negligent, cells would be able to proliferate in the presence of damage but be at risk for developing chromosomal instability and cancer.

## References

[b1] Arias EE, Walter JC (2007). Strength in numbers: preventing rereplication via multiple mechanisms in eukaryotic cells. Genes Dev.

[b2] Arooz T, Yam CH, Siu WY, Lau A, Li KK, Poon RY (2000). On the concentrations of cyclins and cyclin-dependent kinases in extracts of cultured human cells. Biochemistry.

[b3] Bartek J, Lukas J (2007). DNA damage checkpoints: from initiation to recovery or adaptation. Curr Opin Cell Biol.

[b4] Bell SP, Dutta A (2002). DNA replication in eukaryotic cells. Annu Rev Biochem.

[b5] Beucher A, Birraux J, Tchouandong L, Barton O, Shibata A, Conrad S, Goodarzi AA, Krempler A, Jeggo PA, Lobrich M (2009). ATM and Artemis promote homologous recombination of radiation-induced DNA double-strand breaks in G2. EMBO J.

[b6] Boye E, Grallert B (2009). In DNA replication, the early bird catches the worm. Cell.

[b7] Bruno T, De Nicola F, Iezzi S, Lecis D, D'Angelo C, Di Padova M, Corbi N, Dimiziani L, Zannini L, Jekimovs C, Scarsella M, Porrello A, Chersi A, Crescenzi M, Leonetti C, Khanna KK, Soddu S, Floridi A, Passananti C, Delia D, Fanciulli M (2006). Che-1 phosphorylation by ATM/ATR and Chk2 kinases activates p53 transcription and the G2/M checkpoint. Cancer Cell.

[b8] Buscemi G, Perego P, Carenini N, Nakanishi M, Chessa L, Chen J, Khanna K, Delia D (2004). Activation of ATM and Chk2 kinases in relation to the amount of DNA strand breaks. Oncogene.

[b9] Cann KL, Hicks GG (2006). Absence of an immediate G1/S checkpoint in primary MEFs following gamma-irradiation identifies a novel checkpoint switch. Cell Cycle.

[b10] Chan EH, Santamaria A, Silljé HH, Nigg EA (2008). Plk1 regulates mitotic Aurora A function through betaTrCP-dependent degradation of hBora. Chromosoma.

[b11] Chan TA, Hwang PM, Hermeking H, Kinzler KW, Vogelstein B (2000). Cooperative effects of genes controlling the G(2)/M checkpoint. Genes Dev.

[b12] Cheok CF, Bachrati CZ, Chan KL, Ralf C, Wu L, Hickson ID (2005). Roles of the Bloom's syndrome helicase in the maintenance of genome stability. Biochem Soc Trans.

[b13] Chini CC, Chen J (2004). Claspin, a regulator of Chk1 in DNA replication stress pathway. DNA Repair (Amst).

[b14] Chu WK, Hickson ID (2009). RecQ helicases: multifunctional genome caretakers. Nat Rev Cancer.

[b15] Cook JG, Chasse DA, Nevins JR (2004). The regulated association of Cdt1 with minichromosome maintenance proteins and Cdc6 in mammalian cells. J Biol Chem.

[b16] Dannenberg JH, van Rossum A, Schuijff L, te Riele H (2000). Ablation of the retinoblastoma gene family deregulates G(1) control causing immortalization and increased cell turnover under growth-restricting conditions. Genes Dev.

[b17] De Souza CP, Ellem KA, Gabrielli BG (2000). Centrosomal and cytoplasmic Cdc2/cyclin B1 activation precedes nuclear mitotic events. Exp Cell Res.

[b18] Deckbar D, Birraux J, Krempler A, Tchouandong L, Beucher A, Walker S, Stiff T, Jeggo P, Lobrich M (2007). Chromosome breakage after G2 checkpoint release. J Cell Biol.

[b19] Deckbar D, Stiff X Koch B, Reis C, Lobrich M, Jeggo PA (2010). The limitations of the G1-S checkpoint. Cancer Res.

[b20] DePamphilis ML (2003). The ‘ORC cycle’: a novel pathway for regulating eukaryotic DNA replication. Gene.

[b21] Di Leonardo A, Linke SP, Clarkin K, Wahl GM (1994). DNA damage triggers a prolonged p53-dependent G1 arrest and long-term induction of Cipl in normal human fibroblasts. Genes Dev.

[b22] Falck J, Mailand N, Syljuasen RG, Bartek J, Lukas J (2001). The ATM-Chk2-Cdc25A checkpoint pathway guards against radioresistant DNA synthesis. Nature.

[b23] Falck J, Petrini JH, Williams BR, Lukas J, Bartek J (2002). The DNA damage-dependent intra-S phase checkpoint is regulated by parallel pathways. Nat Genet.

[b24] Fekairi S, Scaglione S, Chahwan C, Taylor ER, Tissier A, Coulon S, Dong MQ, Ruse C, Yates JR, Russell P, Fuchs RP, McGowan CH, Gaillard PH (2009). Human SLX4 is a Holliday junction resolvase subunit that binds multiple DNA repair/recombination endonucleases. Cell.

[b25] Fernandez-Capetillo O, Chen HT, Celeste A, Ward I, Romanienko PJ, Morales JC, Naka K, Xia Z, Camerini-Otero RD, Motoyama N, Carpenter PB, Bonner WM, Chen J, Nussenzweig A (2002). DNA damage-induced G2-M checkpoint activation by histone H2AX and 53BP1. Nat Cell Biol.

[b26] Fernet M, Megnin-Chanet F, Hall J, Favaudon V (2010). Control of the G2/M checkpoints after exposure to low doses of ionising radiation: implications for hyper-radiosensitivity. DNA Repair (Amst).

[b27] Fung TK, Poon RY (2005). A roller coaster ride with the mitotic cyclins. Semin Cell Dev Biol.

[b28] Gadbois DM, Lehnert BE (1997). Temporal position of G1 arrest in normal human fibroblasts after exposure to gamma-rays. Exp Cell Res.

[b29] Galgoczy DJ, Toczyski DP (2001). Checkpoint adaptation precedes spontaneous and damage-induced genomic instability in yeast. Mol Cell Biol.

[b30] Ge XQ, Blow JJ (2009). The licensing checkpoint opens up. Cell Cycle.

[b31] Goodarzi AA, Jonnalagadda JC, Douglas P, Young D, Ye R, Moorhead GB, Lees-Miller SP, Khanna KK (2004). Autophosphorylation of ataxia-telangiectasia mutated is regulated by protein phosphatase 2A. EMBO J.

[b32] Hagting A, Karlsson C, Clute P, Jackman M, Pines J (1998). MPF localization is controlled by nuclear export. EMBO J.

[b33] Helleday T, Lo J, van Gent DC, Engelward BP (2007). DNA double-strand break repair: from mechanistic understanding to cancer treatment. DNA Repair (Amst).

[b34] Higa LA, Mihaylov IS, Banks DP, Zheng J, Zhang H (2003). Radiation-mediated proteolysis of CDT1 by CUL4-ROC1 and CSN complexes constitutes a new checkpoint. Nat Cell Biol.

[b35] Hoffelder DR, Luo L, Burke NA, Watkins SC, Gollin SM, Saunders WS (2004). Resolution of anaphase bridges in cancer cells. Chromosoma.

[b36] Hutterer A, Berdnik D, Wirtz-Peitz F, Zigman M, Schleiffer A, Knoblich JA (2006). Mitotic activation of the kinase Aurora-A requires its binding partner Bora. Dev Cell.

[b37] Iliakis G, Wang Y, Guan J, Wang H (2003). DNA damage checkpoint control in cells exposed to ionizing radiation. Oncogene.

[b38] Ip SC, Rass U, Blanco MG, Flynn HR, Skehel JM, West SC (2008). Identification of Holliday junction resolvases from humans and yeast. Nature.

[b39] Jackman M, Lindon C, Nigg EA, Pines J (2003). Active cyclin B1-Cdk1 first appears on centrosomes in prophase. Nat Cell Biol.

[b40] Jackson SP, Bartek J (2009). The DNA-damage response in human biology and disease. Nature.

[b41] Jazayeri A, Falck J, Lukas C, Bartek J, Smith GC, Lukas J, Jackson SP (2006). ATM- and cell cycle-dependent regulation of ATR in response to DNA double-strand breaks. Nat Cell Biol.

[b42] Jeggo PA, Lobrich M (2006). Contribution of DNA repair and cell cycle checkpoint arrest to the maintenance of genomic stability. DNA Repair (Amst).

[b43] Katayama H, Zhou H, Li Q, Tatsuka M, Sen S (2001). Interaction and feedback regulation between STK15/BTAK/Aurora-A kinase and protein phosphatase 1 through mitotic cell division cycle. J Biol Chem.

[b44] Kramer A, Mailand N, Lukas C, Syljuasen RG, Wilkinson CJ, Nigg EA, Bartek J, Lukas J (2004). Centrosome-associated Chk1 prevents premature activation of cyclin-B-Cdk1 kinase. Nat Cell Biol.

[b45] Krempler A, Deckbar D, Jeggo PA, Lobrich M (2007). An imperfect G2M checkpoint contributes to chromosome instability following irradiation of S and G2 phase cells. Cell Cycle.

[b46] Kumagai A, Dunphy WG (2000). Claspin, a novel protein required for the activation of Chk1 during a DNA replication checkpoint response in Xenopus egg extracts. Mol Cell.

[b47] Lee SE, Moore JK, Holmes A, Umezu K, Kolodner RD, Haber JE (1998). Saccharomyces Ku70, mre11/rad50 and RPA proteins regulate adaptation to G2/M arrest after DNA damage. Cell.

[b48] Lindqvist A, Rodríguez-Bravo V, Medema RH (2009). The decision to enter mitosis: feedback and redundancy in the mitotic entry network. J Cell Biol.

[b49] Lindqvist A, van Zon W, Karlsson Rosenthal C, Wolthuis RM (2007). Cyclin B1-Cdk1 activation continues after centrosome separation to control mitotic progression. PLoS Biol.

[b50] Linke SP, Clarkin KC, Wahl GM (1997). p53 mediates permanent arrest over multiple cell cycles in response to gamma-irradiation. Cancer Res.

[b51] Linke SP, Harris MP, Neugebauer SE, Clarkin KC, Shepard HM, Maneval DC, Wahl GM (1997). p53-mediated accumulation of hypophosphorylated pRb after the G1 restriction point fails to halt cell cycle progression. Oncogene.

[b52] Lobrich M, Jeggo PA (2005). The two edges of the ATM sword: co-operation between repair and checkpoint functions. Radiother Oncol.

[b53] Lobrich M, Jeggo PA (2007). The impact of a negligent G2/M checkpoint on genomic instability and cancer induction. Nat Rev Cancer.

[b54] Loffler H, Lukas J, Bartek J, Kramer A (2006). Structure meets function-centrosomes, genome maintenance and the DNA damage response. Exp Cell Res.

[b55] Lopez-Contreras AJ, Fernandez-Capetillo O (2010). The ATR barrier to replication-born DNA damage. DNA Repair (Amst).

[b56] Lu X, Nannenga B, Donehower LA (2005). PPM1D dephosphorylates Chk1 and p53 and abrogates cell cycle checkpoints. Genes Dev.

[b57] Lukas C, Savic V, Bekker-Jensen S, Doil C, Neumann B, Selvlwj Pedersen R, Grefte M, Chan KL, Hickson ID, Bartek J, Lukas J (2011). 53BP1 nuclear bodies form around DNA lesions generated by mitotic transmission of chromosomes under replication stress. Nat Cell Biol.

[b58] Lukas J, Lukas C, Bartek J (2004). Mammalian cell cycle checkpoints: signalling pathways and their organization in space and time. DNA Repair (Amst).

[b59] Macurek L, Lindqvist A, Lim D, Lampson MA, Klompmaker R, Freire R, Clouin C, Taylor SS, Yaffe MB, Medema RH (2008). Polo-like kinase-1 is activated by aurora A to promote checkpoint recovery. Nature.

[b60] Macurek L, Lindqvist A, Medema RH (2009). Aurora-A and hBora join the game of Polo. Cancer Res.

[b61] Mahaney BL, Meek K, Lees-Miller SP (2009). Repair of ionizing radiation-induced DNA double-strand breaks by non-homologous end-joining. Biochem J.

[b62] Mailand N, Bekker-Jensen S, Bartek J, Lukas J (2006). Destruction of Claspin by SCFbetaTrCP restrains Chk1 activation and facilitates recovery from genotoxic stress. Mol Cell.

[b63] Marumoto T, Hirota T, Morisaki T, Kunitoku N, Zhang D, Ichikawa Y, Sasayama T, Kuninaka S, Mimori T, Tamaki N, Kimura M, Okano Y, Saya H (2002). Roles of aurora-A kinase in mitotic entry and G2 checkpoint in mammalian cells. Genes Cells.

[b64] Mazón G, Mimitou EP, Symington LS (2010). Snapshot: Homologous recombination in DNA double-strand break repair. Cell.

[b65] McGarry TJ, Kirschner MW (1998). Geminin, an inhibitor of DNA replication, is degraded during mitosis. Cell.

[b66] Mimitou EP, Symington LS (2009). DNA end resection: many nucleases make light work. DNA Repair (Amst).

[b67] Muñoz IM, Hain K, Declais AC, Gardiner M, Toh GW, Sanchez-Pulido L, Heuckmann JM, Toth R, Macartney T, Eppink B, Kanaar R, Ponting CP, Lilley DM, Rouse J (2009). Coordination of structure-specific nucleases by human SLX4/BTBD12 is required for DNA repair. Mol Cell.

[b68] Munro TR (1970). The relative radiosensitivity of the nucleus and cytoplasm of Chinese hamster fibroblasts. Radiat Res.

[b69] Nakada S, Chen GI, Gingras AC, Durocher D (2008). PP4 is a gamma H2AX phosphatase required for recovery from the DNA damage checkpoint. EMBO Rep.

[b70] Nevis KR, Cordeiro-Stone M, Cook JG (2009). Origin licensing and p53 status regulate Cdk2 activity during G(1). Cell Cycle.

[b71] Nigg EA (2001). Mitotic kinases as regulators of cell division and its checkpoints. Nat Rev Mol Cell Biol.

[b72] Petersen L, Hasvold G, Lukas J, Bartek J, Syljuasen RG (2010). p53-dependent G(1) arrest in 1^st^ or 2^nd^ cell cycle may protect human cancer cells from cell death after treatment with ionizing radiation and Chk1 inhibitors. Cell Prolif.

[b73] Rowles A, Blow JJ (1997). Chromatin proteins involved in the initiation of DNA replication. Curr Opin Genet Dev.

[b74] Sage J, Mulligan GJ, Attardi LD, Miller A, Chen S, Williams B, Theodorou E, Jacks T (2000). Targeted disruption of the three Rb-related genes leads to loss of G(1) control and immortalization. Genes Dev.

[b75] Sancar A, Lindsey-Boltz LA, Unsal-Kacmaz K, Linn S (2004). Molecular mechanisms of mammalian DNA repair and the DNA damage checkpoints. Annu Rev Biochem.

[b76] Sandell LL, Zakian VA (1993). Loss of a yeast telomere: arrest, recovery, and chromosome loss. Cell.

[b77] Seki A, Coppinger JA, Jang CY, Yates JR, Fang G (2008). Bora and the kinase Aurora a cooperatively activate the kinase Plk1 and control mitotic entry. Science.

[b78] Shibata A, Barton O, Noon AT, Dahm K, Deckbar D, Goodarzi AA, Lobrich M, Jeggo PA (2010). Role of ATM and the damage response mediator proteins 53BP1 and MDC1 in the maintenance of G(2)/M checkpoint arrest. Mol Cell Biol.

[b79] Shiotani B, Zou L (2009). Single-stranded DNA orchestrates an ATM-to-ATR switch at DNA breaks. Mol Cell.

[b80] Shreeram S, Demidov ON, Hee WK, Yamaguchi H, Onishi N, Kek C, Timofeev ON, Dudgeon C, Fornace AJ, Anderson CW, Minami Y, Appella E, Bulavin DV (2006). Wip1 phosphatase modulates ATM-dependent signaling pathways. Mol Cell.

[b81] Svendsen JM, Harper JW (2010). GENl/Yenl and the SLX4 complex: Solutions to the problem of Holliday junction resolution. Genes Dev.

[b82] Svendsen JM, Smogorzewska A, Sowa ME, O'Connell BC, Gygi SP, Elledge SJ, Harper JW (2009). Mammalian BTBD12/SLX4 assembles a Holliday junction resolvase and is required for DNA repair. Cell.

[b83] Syljuasen RG (2007). Checkpoint adaptation in human cells. Oncogene.

[b84] Syljuasen RG, Jensen S, Bartek J, Lukas J (2006). Adaptation to the ionizing radiation-induced G2 checkpoint occurs in human cells and depends on checkpoint kinase 1 and Polo-like kinase 1 kinases. Cancer Res.

[b85] Taylor WR, Stark GR (2001). Regulation of the G2/M transition by p53. Oncogene.

[b86] Thomer M, May NR, Aggarwal BD, Kwok G, Calvi BR (2004). Drosophila double-parked is sufficient to induce re-replication during development and is regulated by cyclin E/CDK2. Development.

[b87] Toczyski DP, Galgoczy DJ, Hartwell LH (1997). CDC5 and CKII control adaptation to the yeast DNA damage checkpoint. Cell.

[b88] van Gent DC, Hoeijmakers JH, Kanaar R (2001). Chromosomal stability and the DNA double-stranded break connection. Nat Rev Genet.

[b89] van Vugt MA, Gardino AK, Linding R, Ostheimer GJ, Reinhardt HC, Ong SE, Tan CS, Miao H, Keezer SM, Li J, Pawson T, Lewis TA, Carr SA, Smerdon SJ, Brummelkamp TR, Yaffe MB (2010). A mitotic phosphorylation feedback network connects Cdk1, Plk1, 53BP1, and Chk2 to inactivate the G(2)/M DNA damage checkpoint. PLoS Biol.

[b90] Wahl GM, Linke SP, Paulson TG, Huang LC (1997). Maintaining genetic stability through TP53 mediated checkpoint control. Cancer Surv.

[b91] Warmerdam DO, Kanaar R (2010). Dealing with DNA damage: relationships between checkpoint and repair pathways. Mutat Res.

[b92] Watanabe N, Arai H, Iwasaki J, Shiina M, Ogata K, Hunter T, Osada H (2005). Cyclin-dependent kinase (CDK) phosphorylation destabilizes somatic Wee1 via multiple pathways. Proc Natl Acad Sci USA.

[b93] Watanabe N, Arai H, Nishihara Y, Taniguchi M, Watanabe N, Hunter T, Osada H (2004). M-phase kinases induce phospho-dependent ubiquitination of somatic Wee1 by SCFbeta-TrCP. Proc Natl Acad Sci USA.

[b94] Weinberg RA (1995). The retinoblastoma protein and cell cycle control. Cell.

[b95] Wohlschlegel JA, Dwyer BT, Dhar SK, Cvetic C, Walter JC, Dutta A (2000). Inhibition of eukaryotic DNA replication by geminin binding to Cdt1. Science.

[b96] Yamauchi M, Oka Y, Yamamoto M, Niimura K, Uchida M, Kodama S, Watanabe M, Sekine I, Yamashita S, Suzuki K (2008). Growth of persistent foci of DNA damage checkpoint factors is essential for amplification of G1 checkpoint signaling. DNA Repair (Amst).

[b97] Yao G, Lee TJ, Mori S, Nevins JR, You L (2008). A bistable Rb-E2F switch underlies the restriction point. Nat Cell Biol.

[b98] Yoo HY, Kumagai A, Shevchenko A, Shevchenko A, Dunphy WG (2004). Adaptation of a DNA replication checkpoint response depends upon inactivation of Claspin by the Polo-like kinase. Cell.

[b99] Zetterberg A, Larsson O (1985). Kinetic analysis of regulatory events in G1 leading to proliferation or quiescence of Swiss 3T3 cells. Proc Natl Acad Sci USA.

[b100] Zetterberg A, Larsson O, Wiman KG (1995). What is the restriction point?. Curr Opin Cell Biol.

[b101] Zou L, Elledge SJ (2003). Sensing DNA damage through ATRIP recognition of RPA-ssDNA complexes. Science.

